# Food safety knowledge of undergraduate students at a Canadian university: results of an online survey

**DOI:** 10.1186/s12889-016-3818-y

**Published:** 2016-11-09

**Authors:** Sarah M. Courtney, Shannon E. Majowicz, Joel A. Dubin

**Affiliations:** 1 School of Public Health and Health Systems, University of Waterloo, 200 University Ave. West, Waterloo, Ontario N2L 3G1 Canada; 2 Department of Statistics and Actuarial Sciences, University of Waterloo, Waterloo, Ontario Canada

**Keywords:** Food safety, Food poisoning, Young adult, Cooking, Food handling, Ontario, Canada

## Abstract

**Background:**

Foodborne diseases are an important public health issue, and young adults are an important demographic to target with food safety education. Our objective was to assess the food safety knowledge of undergraduate students at a Canadian university, to identify potential areas for such education.

**Methods:**

In February 2015, we conducted an online survey of 485 undergraduate students at a university in Ontario, Canada. We assessed various food-related factors, including cooking frequency and prior food handling or preparation education. We then modeled the relationship between ‘overall knowledge score’ and the demographic and food skills/cooking experience predictors using multivariable log-binomial regression, to determine factors associated with relatively higher proportions of correct responses.

**Results:**

Respondents were, on average, 20.5 years old, and the majority (64.8 %) lived off campus. Students cooked from basic ingredients infrequently, with 3 in 4 doing so a few times a year to never. Students averaged 6.2 correct answers to the 11 knowledge questions. Adjusting for other important covariates, older age and being a current food handler were associated with relatively higher knowledge, whereas working/volunteering in a hospital and infrequent cooking were associated with relatively lower knowledge. Males in the Faculty of Science had relatively higher knowledge than females in the Faculty of Science, both of whom had relatively higher knowledge than all students in other Faculties. Among students who had never taken a food preparation course, knowledge increased with self-reported cooking ability; however, among students who had taken such a course, knowledge was highest among those with low self-reported cooking ability.

**Conclusions:**

Consistent with other similar studies, students in Faculties outside of the Faculty of Science, younger students, and those who cook infrequently could benefit from food safety education. Supporting improved hand hygiene, in particular clarifying hand washing versus hand sanitizing messages, may also be important. Universities can play a role in such education, including as part of preparing students for work or volunteer placements, or as general support for student health and success.

**Electronic supplementary material:**

The online version of this article (doi:10.1186/s12889-016-3818-y) contains supplementary material, which is available to authorized users.

## Background

Foodborne diseases are an important cause of morbidity and mortality worldwide [1]. In Canada, they cause four million domestically-acquired illnesses each year, affecting one in eight people [2] and costing circa $364 to $455 million [3–6]. Norovirus, *Clostridium* perfringens, *Campylobacter spp.*, and *Salmonella spp.* cause the majority of the foodborne illnesses in Canada [2]. In the province of Ontario, the majority are caused by *Campylobacter spp.*, *Salmonella spp.*, and verotoxin-producing *E. coli* [7], with the greatest population burden linked to *Campylobacter spp.* and *Salmonella spp.* infections [8, 9]. The transmission of these and other foodborne pathogens can be prevented via various food safety initiatives along the farm-to-fork continuum, including on-farm pathogen reduction strategies such as livestock vaccination [10], pasteurization of milk during processing [11], and support for proper food handling and hygiene practices by both workers in food service locations (e.g., [12]) and consumers at home (e.g., [13]).

In the home setting, the likelihood that proper food handling and hygiene practices will be used varies by age. Young adults aged 18 and 29 (as well as older adults aged 65 and older) appear more likely to mishandle food than adults of other ages [14–17]. When observed preparing a meal, young adults performed only 50 % of the recommended food safety behaviours [18], and common food hygiene issues observed in this age group include a lack of food thermometers and having refrigerators and freezers at higher-than-recommended temperatures [19], and inadequate hand washing during food handling [20]. Food mishandling and improper food hygiene practices by young adults may contribute, in part, to the increased incidence of both acute gastrointestinal illness [21] and foodborne disease [22], and the relative increase in the reporting of suspected food poisoning [23], that has been observed in this demographic. Hypothesized reasons for young adults’ poor food handling practices relate to insufficient opportunities for learning safe food handling, including because of increasing consumption of already prepared foods [24], and fewer home economics or other types of food handling and preparation classes in public schools [25]. In addition, many young adults have never held employment involving preparing or serving food, do not possess food safety certification, and have not completed a college course in nutrition, food science, or microbiology [25].

Most assessments of consumer food safety include a measurement of the food safety knowledge of the population of interest. For young adults, food safety knowledge has primarily been investigated in college and university students. Although different study years, populations, and knowledge measurement tools make direct comparisons between studies difficult, most have found that students do not possess the appropriate food safety knowledge to protect themselves from foodborne disease (albeit with better knowledge among students with a health or similar major), as follows. Among students at various United States’ (U.S.) colleges, the average percentage of correct answers to food safety knowledge questions has been measured at 49 % (10.3 correct answers out of 21 questions; health majors [26]), and 60 % (53 out of 89 [27]; and [25]). Knowledge appears to vary by major, for example from an average of 64 % correct answers (12.11 out of 19) for engineering majors to 76 % (14.41 out of 19) for dietetics majors [28], and from 73 % correct answers (10.2 out of 14) for non-health majors, to 84 % (11.8 out of 14) for health majors [29]. Another study, which did not report overall knowledge, found that knowledge varied by question, from 17 % of students knowing the proper temperature for reheating leftovers, to 82 % knowing leftovers should be refrigerated within 2 h [30].

Food safety knowledge also appears to be inadequate among university students outside of the U.S. For example, both female students in Jordan (37.39 out of 81 [31]) and Greek students (6 out of 13 [32]) averaged 46 % correct answers to food safety knowledge questions, while Lebanese students averaged 54 % correct answers [33], and students in Turkey averaged 57 % (11.97 out of 21 [34]). Students in Saudi Arabia averaged 75 % correct answers to 15 knowledge questions [35]. A study of Spanish health sciences students, that did not report overall knowledge, found 50 % of students knew to wash utensils used on raw product before cutting cooked products, and that 85 % knew to wash hands before, during, and after food manipulation [36].

In Canada, food safety among young adults is not well understood, with no studies examining food safety among university students (nor other young adult populations). In Ontario, one recent study in high school students found food safety knowledge, attitudes, and self-reported practices to be poor in this age group [37], and another found that young adults generally have poorer food safety knowledge than other older adults [15]. Given the potential importance of young adults as a demographic to target with food safety education, and that university settings may offer opportunities to provide such education (e.g., via content in relevant academic courses, training prior to co-operative education placements, or within residences or dormitories), the objective of our study was to assess the food safety knowledge of undergraduate students at a Canadian university, including demographic factors associated with food safety knowledge, in order to identify areas for, and groups that may benefit from, possible food safety education.

## Methods

In February 2015, we administered an electronic, cross-sectional survey to undergraduate students at the University of Waterloo, a public research university with a population of ~30,000 undergraduate and ~5500 post-graduate students, located in the City of Waterloo, Ontario, Canada (population ~99,000). Using a random number generator, a sub-set of 5000 undergraduate students was selected, using simple random sampling, from the 29,440 active undergraduate students enrolled at the time of the study. This sub-set size was chosen assuming a 10% response rate, to yield a final target sample size of 500 participants; the sample size was calculated to detect an anticipated difference in the mean number of correct food safety knowledge questions between males and females of 6.5 and 7.0 correct answers out of 11 (s.d. 2.5), with a type I error of 0.05 and a power of 0.80.

An email requesting student participation and containing the link to the survey was sent to the 5000 students by the University’s Registrar’s Office on February 26, 2015. The email provided details about the study and included the researchers’ contact information. A reminder email was sent to all 5000 students by the University’s Registrar’s Office, with the exception of those who explicitly requested no further contact about the study, eight days following the initial invitation.

The electronic survey was conducted in the online platform ‘Hosted in Canada Surveys’ (http://www.hostedincanadasurveys.ca). The survey was open for participation from February 26 to March 12, 2015. On the first page of the web survey, students were again provided study details (including their right to discontinue participating at any time), and students gave informed consent before proceeding to the survey questions. In return for their participation, students had the option to provide an email address to be entered into a draw to win one of four $50 gift cards, to a location of their choice (e.g., gas station, book store). The email addresses were captured in a separate file from the survey data and could not be used to link an email address to an individual’s answers. This study was reviewed and received ethics clearance through a University of Waterloo Research Ethics Committee.

Our questionnaire was a modified version of one previously used to assess food safety in Ontario high school students [37]. Briefly, the original questionnaire was developed by selecting questions from existing, validated questionnaires that assessed, among other items, food safety knowledge across a variety of areas such as hand hygiene, cooking temperatures, and food storage [29, 38–41]. We modified the wording to include ‘undergraduate’ instead of ‘high school’, adjusted response options to be relevant (e.g., included older age categories), and added some undergraduate-specific items such as whether students lived in residence or off campus, and to which Faculty within the University they belonged. The questionnaire (Additional file 1) was designed to take approximately 15 min to complete, and contained a range of questions related to food safety. Questions analysed for this study were the 5 demographic questions, the 5 food skills and cooking experience questions, the 11 food safety knowledge questions for which a correct answer exists (e.g., “what is the most hygienic way to wash your hands?”), and the 1 food safety knowledge question for which correct answers are more nuanced (“where do you think food safety problems are most likely to occur?”). All questions were multiple-choice format, and for one question (“where do you think food safety problems are most likely to occur?”) more than one answer could be selected.

Data were analysed in Stata/SE 14.0 for Mac (StataCorp LP, College Station, Texas). Because missing data were infrequent across all questions (see Results), they were omitted from the analysis of each given question. Differences between demographic characteristics of the study participants versus the overall undergraduate student body were tested using t-tests (for mean ages), and Pearson’s chi-square test, and Fisher’s exact test if necessary (for proportions per sex, Faculty, and co-op versus regular system of study). To compare the average age of study participants to the overall undergraduate population, we used ‘study year’ (available for the undergraduate student body in lieu of age) as a proxy, assuming first year students were 18 years old, second year students were 19 years old, and so on, at the time of the study. Pairwise correlations between correct answers to the 11 individual knowledge questions were calculated.

We assessed the demographic and food skills/cooking experience factors as predictors of responses to the individual knowledge items, using multivariable logistic regression. We included all predictor variables in each model, collapsing multiple-level variables to fewer categories if necessary to avoid empty cells. We then modeled the relationship between the dependent variable ‘overall knowledge score’ (out of 11) and the demographic and food skills/cooking experience predictors using multivariable log-binomial regression. Log-binomial models can be used to estimate prevalence ratios [42–44], for example the relative prevalence of a disease in men versus women; here, we used a log-binomial model to assess the relative proportion of correct answers, out of 11, across levels of our predictor variables. We first started with the full model that included all predictors significantly associated with at least one individual knowledge item, and the 11 two-way interactions between these predictors we hypothesized a priori as being plausible (age by current living arrangement; sex by faculty; self-described cooking ability by current food handler status, previous training, currently working/volunteering in a food service location, frequency of cooking from basic ingredients, and current living arrangement; previous training by current food handler status, and currently working/volunteering in a food service location; current living arrangement by frequency of cooking from basic ingredients; and food handler status by currently working/volunteering in a food service location). We included age as a linear term (based on a graphical examination of the relationship between age and overall knowledge score). To avoid empty cells, for the variable ‘cooking ability’, we merged the two smallest categories, that were the two lowest cooking abilities (‘don’t know how to cook’ and ‘can only cook food when the instructions are on the box’), and for the variable ‘cooking frequency’, we merged the three smallest categories, that were the three most frequent (‘a few times a month’, ‘a few times a week’, and ‘at least once a day’). Any non-significant interaction terms were removed from the model. We then removed all non-significant predictors from the model (retaining any non-significant main effects of significant interaction terms), and assessed each removed variable for potential confounding by re-introducing it to the model, examining any changes in sign, significance, or magnitude of the other model predictors, and retaining any non-significant predictors that had such impacts. For all regression models, because we hypothesized that students within any particular area of study (e.g., within Science, or Arts) might be more similar with respect to food safety knowledge (e.g., due to course content), we adjusted for non-independence of students within the six Faculties using the clustered sandwich estimator of variance [45].

## Results

In total, 491 students completed the survey, yielding a response rate (9.8 %) very close to the expected response rate (10 %); 6 surveys were missing the majority of question responses, resulting in a final sample of 485 participants. Missing data were infrequent; the variable with the most missing data (“have you ever taken a course where you were taught how to prepare food…”) was 95.3 % complete (462/485); details of all variables with missing data are given in Additional file 2. Demographic characteristics of survey respondents, the invited sample, and the undergraduate student body of the University are shown in Table 1. Overall, there were more females and students from the Faculties of Applied Health Sciences and Science, and fewer males and students from the Faculty of Mathematics, in the participating sample of students versus the undergraduate student body as a whole. The average age of study participants (20.5 years; 95 % Confidence Interval [C.I.]: 20.3, 20.6) was slightly older than the approximated average age of the undergraduate student body (19.6 years; *p* < 0.0001). The majority of participants lived off campus (64.8 %; 311/480), followed by living at home (17.5 %; 84/480), and living in traditional-style (10.6 %; 51/480) and suite-style (7.1 %; 34/480) residences; similar data were not available for the undergraduate population.

**Table 1:** Demographic characteristics of study participants, the invited sample, and the University of Waterloo undergraduate population, 2015

Demographic Characteristic	Study participants (*n* = 485)		Invited sample (*n* = 5000)	Undergraduate population (*n* = 29,440)
	Percent (number)	95 % C.I.^a^	Percent (number)	Percent
Gender				
Male	35.0 (167)	**(30.8, 39.4)**	55.1 (2755)	54.5
Female	65.0 (310)	**(60.6, 69.2)**	44.9 (2245)	45.5
Faculty				
Applied Health Sciences	10.2 (49)	**(7.8, 13.3)**	8.1 (405)	7.2
Arts	20.0 (96)	(16.6, 23.8)	21.4 (1070)	21.9
Engineering	23.1 (111)	(19.6, 27.1)	22.3 (1115)	22.3
Environment	8.8 (42)	(6.5, 11.6)	6.8 (340)	7.5
Mathematics	14.6 (70)	**(11.7, 18.0)**	21.6 (1080)	21.3
Science	23.3 (112)	**(19.8, 27.3)**	16.8 (840)	17.0
Other	–	–	3.0 (150)	2.8
System of study				
Co-op	63.1 (301)	(58.7, 67.3)	62.2 (3110)	62.2
Regular	36.9 (176)	(32.7, 41.3)	37.8 (1890)	37.8
Study year				
First	–	–	21.2 (1060)	22.5
Second	–	–	26.3 (1315)	26.6
Third	–	–	25.3 (1265)	24.1
Fourth	–	–	24.1 (1205)	24.0
Fifth	–	–	0.3 (15)	0.3
Non-degree	–	–	2.8 (140)	2.4

^a^Significant differences between study participants and the undergraduate student body are shown in bold

Overall, 10.1 % (49/485) of respondents worked or volunteered in a restaurant, deli, or other food service location; 8.5 % (41/485) in a daycare or other place where they interact with children; 6.2 % (30/485) in a hospital; and 1.9 % (9/485) in a retirement home, nursing home, or long-term care facility. Handling food was not limited to respondents working or volunteering in a restaurant, deli, or other food service location, although these respondents were predominantly food handlers (87.8 %; 43/49). Food handling for the public also occurred by those working or volunteering in a retirement home or long-term care facility (44.4 %; 4/9); a day care or other location for children (29.3 %; 12/41); or in a hospital (16.7 %; 5/30). Overall, 1 in 10 (10.6 %; 51/485) respondents reported currently handling food in commercial or public-serving venues. Roughly 2 in 5 respondents (39.2 %; 190/485) had ever taken a course where they were taught to prepare food, such as a high school food and nutrition class, or food handler certification. Such courses were no more nor less frequent among those currently handling food for the public (43.1 %; 22/51) compared to those not doing so (38.2 %; 166/434; *p* = 0.653).

Students cooked from basic ingredients infrequently, with most doing so a few times a year (40.7 %; 195/479) or never (34.2 %; 164/479), followed by a few times a month (16.7 %; 80/479), a few times a week (4.4 %; 21/479), and at least once a day (4.0 %; 19/479). Self-reported cooking ability was advanced, with most students reporting they can “prepare simple meals if I have a recipe to follow” (50.6 %; 243/480), or “cook almost anything” (39.8 %; 191/480). Relatively fewer students reported they “can do the basics from scratch (like boil an egg or make a grilled cheese sandwich) but nothing more complicated” (6.7 %; 32/480), or “can only cook food when the instructions are on the box” (2.3 %; 11/480), and less than one percent felt they “don’t know how to cook” (0.6 %; 3/480).

When asked where they thought food safety problems were most likely to occur, 5.0 % (24/485) indicated they did not know. The remaining respondents selected homes (70.9 %; 327/461), followed by restaurants (64.6 %; 298/461), food processing plants (52.9 %; 244/461), supermarkets (42.7 %; 197/461), warehouses (40.4 %; 186/461), and farms (31.7 %; 146/461). Most respondents selected one (25.2 %; 116/461), two (19.1 %; 88/461), or three (19.5 %; 91/461) of the possible answers. Associations between the predictor variables and the selection of these items is shown in Table 2.

**Table 2:** Odds ratios (and 95 % Confidence Intervals), for demographic and food skills predictors of answers selected in response to the question “where do you think food safety problems are most likely to occur”, among those not indicating ‘I don’t know’ (*n* = 418); significant predictors are shown in bold

	Homes	Restaurants	Food processing plants	Super-markets	Warehouses	Farms
Age (in years)	1.18 (1.00, 1.40)	1.01 (0.84, 1.22)	1.04 (0.83, 1.30)	0.94 (0.79, 1.10)	**0.86 (0.78, 0.95)**	1.03 (0.89, 1.18)
Male sex (female = referent)	1.45 (0.83, 2.55)	0.65 (0.42, 1.01)	0.68 (0.36, 1.28)	0.92 (0.69, 1.24)	0.90 (0.70, 1.14)	0.68 (0.37, 1.26)
Faculty						
Science	referent					
Applied Health Sciences	**0.72 (0.58, 0.89)**	**0.56 (0.49, 0.63)**	**1.38 (1.16, 1.63)**	**1.39 (1.24, 1.57)**	1.07 (0.98, 1.18)	1.00 (0.81, 1.25)
Arts	**0.83 (0.75, 0.89)**	**0.88 (0.81, 0.95)**	0.98 (0.79, 1.20)	**0.84 (0.77, 0.92)**	1.07 (0.98, 1.16)	**0.73 (0.64, 0.83)**
Engineering	0.95 (0.68, 1.33)	1.02 (0.79, 1.32)	1.05 (0.76, 1.44)	**0.55 (0.46, 0.65)**	**0.66 (0.52, 0.86)**	**0.47 (0.31, 0.70)**
Environment	**0.81 (0.71, 0.94)**	0.93 (0.69, 1.23)	**1.53 (1.22, 1.92)**	**1.31 (1.03, 1.68)**	1.25 (0.95, 1.65)	**0.63 (0.41, 0.96)**
Mathematics	**0.67 (0.50, 0.90)**	**0.67 (0.54, 0.83)**	0.89 (0.63, 1.26)	0.97 (0.77, 1.19)	**0.50 (0.40, 0.62)**	**0.64 (0.50, 0.83)**
Co-op program of study (regular program = referent)	1.21 (0.64, 2.28)	0.94 (0.62, 1.41)	0.76 (0.55, 1.04)	1.12 (0.89, 1.41)	1.19 (0.82, 1.72)	1.15 (0.70, 1.91)
Is a current food handler	0.50 (0.10, 2.45)	1.44 (0.37, 5.62)	1.70 (0.40, 7.33)	**4.72 (3.02, 7.35)**	**5.98 (1.79, 20.0)**	0.89 (0.21, 3.72)
Works or volunteers in a…						
…food service location	3.26 (0.57, 18.6)	1.78 (0.12, 25.3)	0.52 (0.23, 1.16)	**0.18 (0.04, 0.78)**	**0.14 (0.02, 0.71)**	1.10 (0.47, 2.59)
…hospital	**2.25 (1.80, 2.81)**	2.26 (0.62, 8.25)	1.14 (0.36, 3.62)	1.23 (0.87, 1.74)	0.83 (0.55, 1.24)	1.22 (0.81, 1.85)
…daycare/child care facility	1.39 (0.47, 4.05)	0.57 (0.24, 1.33)	1.13 (0.34, 3.72)	**0.37 (0.20, 0.71)**	0.66 (0.44, 1.00)	0.64 (0.35, 1.17)
…long-term care/retirement home facility	2.87 (0.16, 52.7)	omitted due to co-linearity	**0.33 (0.11, 0.97)**	0.55 (0.10, 2.91)	0.93 (0.27, 3.24)	0.23 (0.01, 4.41)
Has ever taken a previous food course	**1.61 (1.06, 2.45)**	1.05 (0.67, 1.57)	0.78 (0.33, 1.85)	1.18 (0.72, 1.94)	0.74 (0.40, 1.35)	0.97 (0.64, 1.47)
Current living arrangement						
Living at home	referent					
Traditional-style residence	0.58 (0.27, 1.25)	1.01 (0.57, 1.77)	1.01 (0.51, 2.00)	0.65 (0.26, 1.62)	0.78 (0.42, 1.46)	1.21 (0.40, 3.62)
Suite-style residence	0.67 (0.24, 1.87)	**2.10 (1.11, 3.95)**	1.75 (0.98, 3.11)	0.96 (0.63, 1.47)	0.77 (0.27, 2.21)	2.30 (0.79, 6.74)
Living off campus	0.74 (0.50, 1.09)	**1.56 (1.07, 2.27)**	0.89 (0.61, 1.30)	1.01 (0.55, 1.86)	1.02 (0.64, 1.63)	1.09 (0.41, 2.91)
Frequency of cooking from basic ingredients						
Never or a few times a year	referent					
A few times a month	**0.48 (0.25, 0.94)**	0.97 (0.51, 1.82)	1.69 (0.68, 4.21)	0.65 (0.26, 1.62)	1.45 (0.78, 2.68)	1.27 (0.69, 2.32)
A few times a week or more	1.15 (0.49, 2.69)	1.21 (0.54, 2.67)	**0.54 (0.31, 0.94)**	0.96 (0.63, 1.47)	1.40 (0.60, 3.25)	0.84 (0.27, 2.63)
Good self-described cooking ability^a^	1.19 (0.49, 2.69)	0.94 (0.57, 1.57)	0.67 (0.34, 1.32)	1.31 (0.92, 1.86)	0.78 (0.37, 1.63)	0.96 (0.35, 2.62)

^a^Those reporting the ability to cook the basics from scratch, prepare simple meals from a recipe, or cook almost anything (referent: those reporting they don’t know how to cook, or that they can only cook food when the instructions are on the box)

The knowledge question for which the correct answer was selected most frequently was the description of microorganisms (Table 3); interestingly, although most incorrect answers related to a possible increase in foodborne disease risk (e.g., cutting meat open in lieu of using a food thermometer), one incorrect answer (storing leftovers for 1–2 days, instead of 3–4 days) did not. Relationships between the predictor variables and correct answers for the 11 knowledge questions are shown in Tables 4 and 5; working or volunteering in a long-term care or retirement facility was the only variable not associated with a correct answer for any of the knowledge questions. No knowledge questions had correct answers perfectly predicted by, or nested within, other answers. All pairwise correlations between correct responses for the individual knowledge questions were less than or equal to 0.170. Knowing that a food thermometer is the best way to check hamburger doneness was correlated with knowing how long leftover foods should be heated (*r* = 0.168; 95 % C.I. 0.075, 0.248), and what to do with accidentally thawed meat (*r* = 0.163; 95 % C.I. 0.081, 0.253). What to do with accidentally thawed meat was also correlated with knowing how to safely store a hot meal to be eaten several hours later (*r* = 0.170; 95 % C.I. 0.083, 0.255).

**Table 3:** Percent of University of Waterloo undergraduate student respondents (*n* = 485) selecting the correct answer, and the most frequently selected incorrect answer, to food safety knowledge questions

Question	Percent of Students Selecting a Given Answer			
	Correct Answer	%	Most Frequent Incorrect Answer	%
What are microorganisms?	Small living things that are too small to be seen with our eyes	96.8	Poisons that can contaminate our food and water	2.3
Which of the following is considered the most important way to prevent food poisoning?	Keep foods refrigerated until it’s time to cook or serve them	84.3	Clean kitchen counters with sanitizing solutions weekly	11.7
Chilling or freezing eliminates harmful germs	False	77.0	True	23.0
Which is the most hygienic way to wash your hands?	Run water, moisten hands, apply soap, rub hands together for 20 s, rinse hands, dry hands	71.5	Apply soap, rub hands together for 20 s, rinse hands under water, dry hands, apply sanitizer	15.4
If a family member is going to be several hours late for a hot meal, how should you store the meal to keep it safe until this person is ready to eat it?	Store it in the refrigerator and reheat it when the person is ready to eat it	65.7	Store it a warm oven until the person is ready to eat it	26.9
Imagine your electricity went off and the meat, chicken, and/or seafood in your freezer thawed and felt warm. What should you do?	Throw them away	56.0	See how they smell or look before deciding what to do	30.1
Which method is the best way of determining whether hamburgers are cooked enough?	Measure the temperature with a food thermometer	51.4	Cut one to check the colour of the meat inside	35.4
How long should leftovers be stored in the refrigerator?	3–4 days	37.2	1–2 days	36.3
All foods (except whole poultry) are considered safe when cooked to an internal temperature of:	165^°^ F (74^°^ C)	41.6	150^°^ F (66^°^ C)	31.0
To prevent food poisoning, how long should leftover foods be heated?	Until they are boiling hot	31.8	Just until they are hot, but not too hot to eat right away	43.4
Which procedure for cleaning kitchen counter is best?	Wash with a detergent, rinse, then wipe with a sanitizing solution	24.0	Wipe with a sanitizing solution, then rinse with clean water and wipe dry	44.2

**Table 4:** Odds ratios (and 95 % Confidence Intervals), for demographic and food skills predictors of correct answers for individual food safety knowledge questions, answered correctly by more than 50 % of respondents (*n* = 485); significant predictors are shown in bold

	Defining Micro-organism^a^	Preventing Food Poisoning^b^	Chilling/Freezing^c^	Hand Washing^d^	Storing Meals to Eat Later^e^	Accidental Freezer Thawing^f^	Determining Burger Doneness^g^
Age (in years)	0.95 (0.73, 1.24)	1.01 (0.83, 1.22)	1.13 (0.96, 1.33)	1.01 (0.91, 1.12)	0.98 (0.75, 1.28)	1.05 (0.91, 1.21)	1.11 (0.92, 1.34)
Male sex (female = referent)	**2.21 (1.47, 3.32)**	1.05 (0.68, 1.63)	1.04 (0.52, 2.05)	0.95 (0.61, 1.50)	1.68 (0.87, 3.24)	1.05 (0.62, 1.77)	0.70 (0.40, 1.25)
Faculty							
Science	referent						
Applied Health Sciences	**0.17 (0.087, 0.33)**	**6.96 (4.95, 9.79)**	**0.70 (0.50, 0.98)**	**0.75 (0.59, 0.95)**	0.73 (0.51, 1.04)	**0.71 (0.62, 0.81)**	1.15 (0.82, 1.61)
Arts	0.08 (0.59, 1.01)	1.27 (0.98, 1.64)	**0.89 (0.80, 0.98)**	**0.53 (0.47, 0.60)**	**2.34 (2.00, 2.74)**	**1.24 (1.12, 1.37)**	**0.76 (0.63, 0.92)**
Engineering	**0.12 (0.04, 0.37)**	0.57 (0.29, 1.11)	0.88 (0.54, 1.41)	**0.56 (0.35, 0.91)**	**0.65 (0.46, 0.91)**	**0.56 (0.45, 0.69)**	0.83 (0.60, 1.15)
Environment	**0.36 (0.14, 0.92)**	1.24 (0.98, 1.57)	0.97 (0.77, 1.22)	**0.76 (0.68, 0.86)**	**1.63 (1.24, 2.15)**	0.91 (0.77, 1.07)	1.25 (0.88, 1.78)
Mathematics	**0.17 (0.08, 0.37)**	0.92 (0.56, 1.51)	1.17 (0.88, 1.57)	**0.33 (0.23, 0.46)**	**0.51 (0.37, 0.70)**	**0.65 (0.56, 0.76)**	**1.17 (1.01, 1.35)**
Co-op program of study (regular program = referent)	1.12 (0.23, 5.34)	0.97 (0.35, 2.68)	1.05 (0.55, 2.00)	0.77 (0.34, 1.71)	1.26 (0.86, 1.84)	1.08 (0.77, 1.51)	1.26 (0.82, 1.94)
Is a current food handler	4.57 (0.10, 215)	**5.41 (1.94, 15.1)**	0.48 (0.12, 1.89)	3.22 (0.81, 12.7)	0.52 (0.19, 1.45)	**3.73 (1.91, 7.30)**	0.58 (0.14, 2.38)
Works or volunteers in a…							
…restaurant or other food service location	0.23 (0.001, 41.1)	0.20 (0.03, 1.27)	3.64 (0.46, 28.8)	0.565 (0.17, 1.79)	0.80 (0.45, 1.44)	0.83 (0.31, 2.17)	2.78 (0.25, 30.5)
…hospital	0.46 (0.05, 4.14)	0.93 (0.14, 6.20)	0.65 (0.36, 1.20)	**0.41 (0.27, 0.63)**	0.95 (0.33, 2.75)	0.99 (0.61, 1.61)	0.91 (0.43, 1.91)
…daycare/child care facility	**0.27 (0.11, 0.67)**	0.64 (0.23, 1.77)	0.72 (0.25, 2.07)	0.74 (0.27, 1.98)	1.82 (0.74, 4.49)	0.88 (0.41, 1.90)	0.77 (0.30, 1.97)
…long-term care/retirement home facility	omitted due to co-linearity	1.10 (0.10, 12.3)	2.97 (0.30, 29.9)	2.378 (0.50, 11.4)	2.53 (0.49, 12.9)	1.53 (0.23, 10.1)	1.33 (0.17, 10.5)
Has ever taken a previous food course	1.71 (0.53, 5.54)	1.20 (0.65, 2.24)	1.22 (0.73, 2.04)	0.94 (0.46, 1.92)	**1.60 (1.09, 2.36)**	0.71 (0.48, 1.05)	1.45 (0.98, 2.15)
Current living arrangement							
Living at home	referent						
Traditional-style residence	1.03 (0.24, 4.43)	0.85 (0.30, 2.41)	0.84 (0.38, 1.86)	**0.49 (0.27, 0.88)**	0.75 (0.33, 1.71)	1.17 (0.37, 3.71)	1.46 (0.78, 2.71)
Suite-style residence	1.26 (0.11, 15.1)	0.24 (0.06, 1.01)	0.62 (0.24, 1.60)	1.24 (0.52, 2.95)	**0.38 (0.18, 0.87)**	**0.52 (0.28, 0.96)**	1.43 (0.60, 3.42)
Living off campus	0.92 (0.21, 4.10)	0.71 (0.28, 1.76)	0.78 (0.48, 1.26)	**0.60 (0.41, 0.87)**	0.64 (0.40, 1.03)	0.77 (0.60, 1.00)	0.72 (0.31, 1.68)
Frequency of cooking from basic ingredients							
Never or a few times a year	referent						
A few times a month	**0.44 (0.21, 0.90)**	0.65 (0.30, 1.43)	0.62 (0.30, 1.29)	0.86 (0.63, 1.18)	0.93 (0.56, 1.64)	1.00 (0.70, 1.42)	**0.55 (0.31, 0.95)**
A few times a week or more	1.90 (0.57, 6.31)	0.61 (0.23, 1.61)	0.87 (0.31, 2.45)	0.98 (0.32, 2.98)	**0.60 (0.46, 0.78)**	1.25 (0.55, 2.84)	0.77 (0.39, 1.52)
Good self-described cooking ability^h^	1.43 (0.22, 9.34)	1.16 (0.75, 1.86)	1.14 (0.40, 3.22)	1.19 (0.79, 1.79)	0.99 (0.68, 1.44)	0.97 (1.14, 3.52)	**2.92 (1.39, 6.14)**

^a^What are microorganisms?

^b^Which of the following is considered the most important way to prevent food poisoning?

^c^Chilling or freezing eliminates harmful germs (true or false)

^d^Which is the most hygienic way to wash your hands?

^e^If a family member is going to be several hours late for a hot meal, how should you store the meal to keep it safe until this person is ready to eat it?

^f^Imagine your electricity went off and the meat, chicken, and/or seafood in your freezer thawed and felt warm. What should you do?

^g^Which method is the best way of determining whether hamburgers are cooked enough?

^h^Those reporting the ability to cook the basics from scratch, prepare simple meals from a recipe, or cook almost anything (referent: those reporting they don’t know how to cook, or that they can only cook food when the instructions are on the box)

**Table 5:** Odds ratios (and 95 % Confidence Intervals), for demographic and food skills predictors of correct answers for individual food safety knowledge questions, answered correctly by fewer than 50 % of respondents (*n* = 485); significant predictors are shown in bold

	Leftover Storage Time^a^	Internal Cooking Temperature^b^	Reheating Leftovers^c^	Cleaning Counters^d^
Age (in years)	0.94 (0.81, 1.09)	1.05 (0.91, 1.22)	**1.39 (1.23. 1.56)**	1.19 (0.90, 1.56)
Male sex (female = referent)	0.88 (0.45, 1.73)	**1.69 (1.26, 2.26)**	0.87 (0.58, 1.30)	0.99 (0.61, 1.60)
Faculty				
Science		referent		
Applied Health Sciences	0.87 (0.65, 1.17)	**0.65 (0.53, 0.80)**	**0.85 (0.74, 0.98)**	0.99 (0.73, 1.35)
Arts	**0.59 (0.51, 0.67)**	**0.50 (0.45, 0.56)**	**0.51 (0.44, 0.59)**	**0.78 (0.63, 0.96)**
Engineering	0.95 (0.49, 1.86)	**0.47 (0.36, 0.62)**	0.76 (0.55, 1.05)	**1.58 (1.35, 1.85)**
Environment	**1.72 (1.34, 2.21)**	**0.29 (0.26, 0.33)**	**0.58 (0.46, 0.74)**	1.24 (0.92, 1.68)
Mathematics	0.52 (0.34, 0.81)	**0.67 (0.56, 0.81)**	**1.41 (1.08, 1.85)**	1.17 (0.97, 1.41)
Co-op program of study (regular program = referent)	1.00 (0.43, 2.37)	0.80 (0.51. 1.24)	1.12 (0.70, 1.80)	**0.73 (0.57, 0.94)**
Is a current food handler	5.46 (0.91, 32.8)	0.84 (0.38, 1.84)	0.31 (0.03, 2.89)	**4.85 (1.41, 16.6)**
Works or volunteers in a…				
…food service location	**0.12 (0.03, 0.58)**	1.92 (0.47, 7.90)	3.55 (0.57, 22.3)	0.35 (0.10, 1.25)
…hospital	0.59 (0.34, 1.02)	1.08 (0.47, 2.48)	0.65 (0.20, 2.11)	0.61 (0.21, 1.79)
…daycare/child care facility	0.88 (0.41, 1.88)	**2.18 (1.12, 4.25)**	0.89 (0.31, 2.55)	0.77 (0.28, 2.10)
…long-term care/retirement home facility	2.66 (0.41, 17.5)	0.74 (0.17, 3.17)	0.81 (0.37, 1.76)	0.93 (0.20, 4.35)
Has ever taken a previous food course	1.37 (0.96, 1.97)	0.93 (0.57, 1.52)	0.95 (0.75, 1.20)	**0.66 (0.48, 0.91)**
Current living arrangement				
Living at home	referent			
Traditional-style residence	**0.45 (0.24, 0.86)**	0.49 (0.06, 3.80)	1.80 (0.92, 3.51)	2.10 (0.63, 6.96)
Suite-style residence	**0.31 (0.10, 0.97)**	1.77 (0.70, 4.51)	2.80 (0.94, 8.40)	1.75 (0.63, 4.84)
Living off campus	0.90 (0.72, 1.11)	1.36 (0.85, 2.17)	0.98 (0.50, 1.95)	0.92 (0.39, 2.21)
Frequency of cooking from basic ingredients				
Never or a few times a year	referent			
A few times a month	1.93 (0.89, 4.18)	1.04 (0.63, 1.69)	0.88 (0.63, 1.22)	0.85 (0.68, 1.05)
A few times a week or more	1.57 (0.78, 3.18)	**2.08 (1.33, 3.25)**	**0.50 (0.31, 0.81)**	0.46 (0.09, 2.29)
Good self-described cooking ability^e^	1.50 (0.44, 5.18)	**3.54 (1.88, 6.67)**	0.82 (0.53, 1.27)	1.98 (0.55, 7.10)

^a^How long should leftovers be stored in the refrigerator?

^b^All foods (except whole poultry) are considered safe when cooked to an internal temperature of (select one)

^c^To prevent food poisoning, how long should leftover foods be heated?

^d^Which procedure for cleaning kitchen counter is best?

^e^Those reporting the ability to cook the basics from scratch, prepare simple meals from a recipe, or cook almost anything (referent: those reporting they don’t know how to cook, or that they can only cook food when the instructions are on the box)

Participants averaged 6.2 (s.d. = 1.79; min = 1, max = 11; median = 6) correct answers to the 11 knowledge questions. The final multivariable log-binomial regression model, showing adjusted prevalence ratios for significant predictors, is given in Table 6. Two factors were associated with a relatively higher knowledge score; adjusting for the other model variables, for each additional year of age, the estimated proportion of correct answers was 1.02 greater, and the estimated proportion of correct answers was 1.11 times higher in students who were current food handlers than those who were not. Two factors were associated with relatively lower knowledge scores; adjusting for the other model variables, the estimated proportion of correct answers was 1.09 times lower (95 % C.I. 1.00, 1.19; *p* = 0.040; i.e., 0.92 times higher) in students who worked or volunteered in a hospital than those who did not, and the estimated proportion of correct answers was 1.05 times lower (95 % C.I. 1.00, 1.10; *p* = 0.033; i.e., 0.95 times higher) in students who reported cooking only a few times a year, versus those who never cook.

**Table 6:** Relative proportions (i.e., prevalence ratios) of the number of correct answers for 11 food safety knowledge questions, with 95 % Confidence Intervals (CIs), for the significant demographic and food skills predictors (*n* = 485); significant predictors are shown in bold

	Relative Proportion	*P*-value	95 % CI
Age (in years)	**1.02**	**0.045**	**1.00** ^**a**^ **, 1.05**
Male sex (female = referent)	0.98	0.248	0.94, 1.02
Faculty of Science (all other Faculties = referent)	**1.06**	**<0.001**	**1.04, 1.07**
Male Sex*Faculty of Science	**1.13**	**<0.001**	**1.07, 1.19**
Is a current food handler	**1.11**	**0.010**	**1.02, 1.19**
Works or volunteers in a hospital	**0.91**	**0.040**	**0.84, 1.00**
Frequency of cooking from basic ingredients (never = referent)			
A few times a year	**0.95**	**0.033**	**0.91, 1.00**
A few times a month or more	0.96	0.237	0.90, 1.03
Has ever taken a previous food course	**1.58**	**<0.001**	**1.39, 1.79**
Self-described cooking ability (don’t know how to, or can cook when instructions are on box = referent)			
Can cook the basics from scratch (e.g., boil an egg)	1.15	0.098	0.98, 1.34
Can prepare simple meals from a recipe	**1.33**	**0.003**	**1.10, 1.60**
Can cook almost anything	**1.44**	**<0.001**	**1.18, 1.76**
Has ever taken a previous food course*self-described cooking ability			
Can cook the basics from scratch (e.g., boil an egg)	**0.68**	**0.002**	**0.53, 0.86**
Can prepare simple meals from a recipe	**0.65**	**<0.001**	**0.57, 0.75**
Can cook almost anything	**0.61**	**<0.001**	**0.55, 0.69**

^a^lower bound of confidence interval = 1.001

**Fig. 1:**
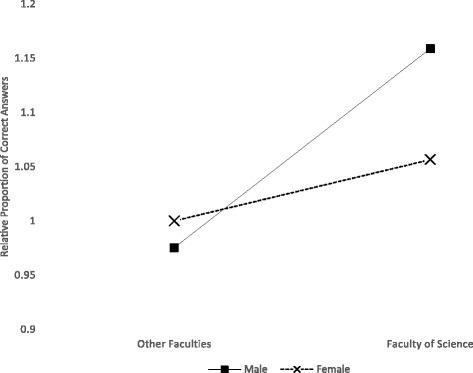
The relative proportion of correct answers to 11 food safety knowledge questions, by sex and Faculty, among University of Waterloo undergraduate student respondents (*n* = 485), adjusting for other demographic and food skills/experience predictors

**Fig. 2:**
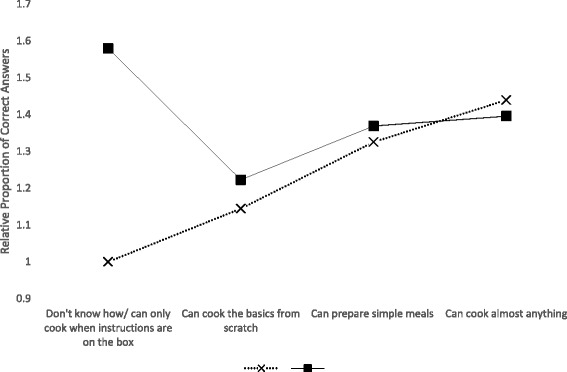
The relative proportion of the number of correct answers to 11 food safety knowledge questions, by self-reported cooking ability and whether or not respondents had ever taken a prior course in which they were taught to prepare or handle food, among University of Waterloo undergraduate student respondents (*n* = 485), adjusting for other demographic and food skills/experience predictors

There was a significant interaction between being in the Faculty of Science and being male (Fig. 1). Adjusting for the other model variables, the estimated proportion of correct answers was 1.16 (95 % C.I. 1.04, 1.29; *p* < 0.001) and 1.06 times higher (95 % C.I. 1.04, 1.07; *p* < 0.001) for males and females in the Faculty of Science, respectively, compared to females in the other Faculties, although the proportions of correct answers for males and for females in other Faculties were similar. There was also a significant interaction between self-reported cooking ability and whether or not the student had ever taken a course in which they were taught to prepare or handle food (Fig. 2). Adjusting for the other model variables, in students who had never taken a previous course, the proportion of correct answers increased as self-reported cooking ability increased, such that the estimated proportion of correct answers was 1.44 times higher (95 % C.I. 1.18, 1.76; *p* < 0.001) for those who reported they ‘can cook almost anything’ versus those who reported they don’t know how to cook or can only cook ‘when the instructions are on the box’. Among students who had taken a course in which they had been taught to prepare or handle food, the estimated proportion of correct answers was highest among those who reported they don’t know how to cook or can only cook ‘when the instructions are on the box’.

## Discussion

We conducted an online survey of the food safety knowledge of undergraduate students at the University of Waterloo, a public research university with a population of ~30,000 undergraduate students located in Ontario, Canada. Overall, we found knowledge to be poor, and generally comparable to that of other college and university students worldwide (Table 7); knowledge was slightly higher, however, than that of high school students (who averaged 47 % correct responses), measured during the same time frame and in the same province [37]. In this study, our university students answered an average of 56 % of the knowledge questions correctly. For all but one question (leftovers, discussed below), the most frequently selected incorrect answer related to an increased foodborne disease risk. That students’ inaccurate food safety knowledge may increase foodborne disease risk is important, because although students reported cooking from basic ingredients infrequently (with 3 in 4 students reporting cooking only a few times a year or never), the majority (65 %) lived off-campus and presumably handled food for their own consumption in some capacity. As well, 1 in 10 students reported currently handling food for the public (including via working or volunteering in food service premises, day cares, long-term care facilities, and hospitals). Although those currently handling food did provide relatively more correct answers than did non-food handlers, the types of correct answers more frequent among food handlers pertained to refrigeration, thawing, and cleaning counters. Food handlers were no more knowledgeable about other food safety items, including correct hand washing and thermometer use, and thus still may represent a potential risk to others.

**Table 7:** Proportion of college and university students correctly answering food safety knowledge questions, compared across commonly used questions from an existing, validated food safety knowledge instrument [40], by study year and country

Food safety knowledge question, as worded in this study (showing subscale^a^, and question number from original questionnaire reference [40])	This study: undergraduate students at the University of Waterloo, Canada, 2015 (*n* = 485)	Undergraduate students of the Lebanese American University, Lebanon, 2013 (*n* = 1172) [33]	Undergraduate students attending a major American university, 2013 (*n* = 786) [26]	University students at the Aristotle University of Thessaloniki, Greece, 2010 (*n* = 837) [32]	Female college students living at private and university dorms, Irbid city, Jordan, 2009 (*n* = 867) [31]
Which procedure for cleaning kitchen counter is best? (CC, 5)	24.0 %	78.7 %	27 %	32.0 %	31 %
Which is the most hygienic way to wash your hands? (CC, 7)	71.5 %	–	55 %	–	51 %
Imagine your electricity went off and the meat, chicken, and/or seafood in your freezer thawed and felt warm. What should you do? (ST, 4)	56.0 %	–	–	–	20.1 %
Which of the following is considered the most important way to prevent food poisoning? (ST, 5)	84.3 %	–	71 %	51.3 %	58.4 %
If a family member is going to be several hours late for a hot meal, how should you store the meal to keep it safe until this person is ready to eat it? (ST, 8)	65.7 %	–	–	–	49.8 %
All foods (except whole poultry) are considered safe when cooked to an internal temperature of: (ST, 9)	41.6 %	–	–	20.7 %	33 %
Which method is the best way of determining whether hamburgers are cooked enough? (ST, 10)	51.4 %	–	53 %	–	8.2 %
To prevent food poisoning, how long should leftover foods be heated? (ST, 12)	31.8 %	–	–	–	36.9 %
Chilling or freezing eliminates harmful germs (FR, 1)	77.0 %	64.0 %	60 %	78.3 %	52.2 %

^a^ *CC* cross contamination prevention/disinfection procedures scale, *ST* safe times/temperatures for cooking/storing food scale, *FR* foods that increase the risk of foodborne disease scale

Here, roughly 70 % of students knew the correct way to wash hands, which is higher than has been previously reported in other college/university populations where studies have used the same survey question (Table 7). Of concern, however, is that those working or volunteering in a hospital, as well as those living in residence or off-campus, were half as likely to know the correct way to wash hands (versus those not working/volunteering in a hospital, and those living at home, respectively), adjusting for all other factors considered. The incorrect options for the hand washing multiple-choice question all involved the use of hand sanitizers. That students selected options with sanitizers, including sanitizer use without soap and water, suggests that students may not understand the mechanics and purpose of hand washing versus hand sanitizing. Further exploration of students’ understanding of various hand hygiene activities, including perceived advantages of both washing and sanitizing, may be important in targeting hand hygiene messages; such messages should also consider that university students appear to be more motivated by social norms around acceptable hand hygiene behaviours than by scientific knowledge [46]. Given that only 26 % of students at a Texas university washed their hands adequately – and 27 % did not wash their hands at all – when using campus restroom facilities [47], and that poor hand hygiene among university students has been linked to increased infectious diseases, medical visits, and absence from class [48], supporting improved hand hygiene among students may be an important way for academic institutions to improve student health and contribute to academic success. Thus, colleges and universities should consider providing hand hygiene education to students, perhaps prior to co-operative education or volunteer placements, or prior to leaving residences to move to off-campus locations (in this population, typically at the end of the first or second year of undergraduate studies).

In this study, males in the Faculty of Science had relatively higher knowledge than females in the Faculty of Science, both of whom had relatively higher knowledge than all students in other Faculties (including the Faculty of Applied Health Sciences). Our finding is in line with results from other studies, where food safety knowledge has been found to be higher among health- and physical science-oriented students [28, 29, 31–33], although one recent study found no difference between health and non-health students [26]. The link between food safety knowledge and a science-oriented program of study at university is intuitive, as many such programs require courses in microbiology, where course content and bench work requirements may expose students to concepts of hygiene, contamination prevention, and microbial growth and inactivation. Here, many of the degree programs in the Faculty of Science have a microbiology course requirement; in comparison, none of the degree programs in Applied Health Sciences require microbiology.

Our finding above differs, however, from the many studies which have found higher food safety knowledge in females versus males [14, 15, 25, 28, 29, 32, 33]. It is unclear why our study’s findings are in contrast, and variation in study populations, timeframes, and specific knowledge questions may have influenced this result. However, another potential explanation is that our estimates are adjusted for other factors, including self-reported cooking ability and cooking frequency, and include the interaction between sex and area of study, which may have been unadjusted for previous studies’ findings, particularly older studies that relied univariable and bivariable analyses. Another study, by Sharif et al in 2010 [35], found that male health students and female humanities students had higher knowledge than male humanities students at Taif University in Jordan (there were no female health students), which is more closely aligned with our findings. Future studies should explore in more detail the nature of the relationship between sex and food safety knowledge among university students, and all studies of food safety knowledge, regardless of target population, should ensure that important confounders are measured and accounted using multivariable analyses.

Here, we found an interesting interaction between having taken a previous course in which students were taught to handle or prepare food (e.g., food handler training, home economics courses), and students’ self-described cooking ability. Our finding that food safety knowledge was higher in those who had taken a previous food course versus those who had not, among those who reported they essentially don’t know how to cook, is intuitive. Similarly, our finding that food safety knowledge increased with self-described cooking ability, among those who have not taken a previous food course, is also intuitive. However, our finding that – among those who had previously taken a food course – food safety knowledge declined, and then only marginally rose, as self-described cooking ability increased from not knowing how to cook, to basic cooking abilities, and then to excellent cooking abilities, has not been previously reported. This finding bears further investigation, and future studies may wish to investigate how development of food preparation skills may somehow supersede or override previously learned knowledge, as well as how individuals of different self-described cooking abilities operationalize food safety knowledge into practice during food preparation.

Among the undergraduate students in this study, 37 % correctly identified that leftovers can be stored in the refrigerator for 3 to 4 days, whereas another 36 % thought leftovers could only be stored for 1 to 2 days. If indeed students are throwing leftovers away prematurely as a result, this represents a potential area for education, particularly given that food insecurity is an issue among both University of Waterloo students (e.g., [49, 50]) and Canadian university students in general [51–54]. However, caution must be used in crafting such messages, since proper handling of leftovers involves not only the amount of time they can be retained in the refrigerator, but also their proper reheating. Only 32 % of students in this study correctly identified that leftovers need to be reheated until they are boiling hot, with the rest selecting inadequate reheating options including no reheating. Thus, messages about the proper handling of leftovers that stress both a three-to-four day storage time and that leftovers must also be fully reheated until boiling (or better, until the internal temperature reaches 74 °C), may be a way to address food safety, food security, and food waste among students.

This study is subject to several limitations inherent in food safety knowledge surveys, most notably the potentially limited generalizability of findings to students outside the studied institution, and the limited number of knowledge questions we were able to include. In addition, our use of multiple choice questions, that by design provide respondents with the correct answer option (versus open-ended formats), may have led to an overestimate of students’ true knowledge; how student performance on multiple choice questions relates to their true food safety knowledge merits further investigation. Our use of an online survey with recruitment via mass email may have influenced the types of students responding to the survey. Here, our respondents included more females and students from the Faculties of Applied Health Science and Science, compared to undergraduate students overall. Given our finding that being in the Faculty of Science was associated with greater knowledge (adjusting for other factors), the mean food safety knowledge score reported for our student sample likely overestimates the mean food safety knowledge of the undergraduate student body as a whole. A final potential limitation is our exclusion of missing data. This said, missing data were infrequent (i.e., except for “previous food course” missing at 4.74 %, no other variable had missing percentage higher than 1.65 %, and most were at 0 %), and hence unlikely to result in much bias in our reported results.

Despite these limitations, we identified several important areas for targeted food safety messages, and our findings are generally in line with those for other similar populations. Students in Faculties other than the Faculty of Science may benefit from general food safety education, whether through courses, or via extra-curricular activities. It may also be useful to target education to younger students, and to those who cook infrequently, and to time such education so it occurs while students are in residence, and prior to co-operative education or volunteer placements. Providing general support for improved hand hygiene across the undergraduate population as a whole, and providing detailed hand washing versus hand sanitizing messages to those who work or volunteer in hospital settings, may also be important. Educating students about proper handling of leftovers, making sure to combine messages about storage times with proper reheating, may be a way to address food safety in tandem with food security in this undergraduate population.

## Conclusion

In 1998, Unklesbay et al. [28] published one of the first explorations of food safety among college students, and concluded with a call for improved food safety education, specifically that “…the role of [food-related educators] should be expanded to include all college disciplines, especially as the majority of the U.S. population is one or more generations removed from direct experiences on farms and ranches…” and that “…students and the public need to be empowered to make informed decisions.” In the 18 years since this call to action, there have been many assessments of food safety knowledge, attitudes, and practices among college and university students in a variety of settings and countries [14, 20, 25, 26, 28, 31–33, 35], including several that have evaluated the effectiveness of different interventions aimed at improving these factors [29, 55]. Despite this, food safety among college and university students appears to still be an important yet inadequately addressed issue, as evidenced in part by our findings. Here we found that students in Faculties outside of the Faculty of Science, younger students, and those who cook infrequently could benefit from food safety education, and that supporting improved hand hygiene, in particular clarifying hand washing versus hand sanitizing messages, may also be important. Academic institutions should consider their role in providing both general and targeted information, particularly if such provision can be viewed as a key part of preparing students for work or volunteer placements, or as part of supporting student health and success in general.

## Additional files

Additional file 1: Study questionnaire. (PDF 367 kb)
Additional file 2: Variables with Missing Data. (DOCX 51 kb)
