# Supplementary material for: Food safety knowledge of undergraduate students at a Canadian university: results of an online survey
Source: BMC Public Health. 2016 Nov 9;16:1147. doi: 10.1186/s12889-016-3818-y (PMC5103385; doi:10.1186/s12889-016-3818-y)
Supplement: Additional file 1: — Study questionnaire. (PDF 367 kb) [file 12889_2016_3818_MOESM1_ESM.pdf]

Food Safety Knowledge, Behaviours, and Beliefs among Undergraduate Students at the University of Waterloo

UNIVERSITY OF
WATERLOO

University of Waterloo School of Public Health and Health Systems

Consent to Participate in Research ORE # 20347

Food Safety Knowledge, Behaviours and Beliefs in Undergraduate Students at the University of Waterloo

You are being asked to participate in a research study led by Sarah Courtney, BSc candidate, and advisor Dr. Shannon Majowicz from the School of Public Health and Health Systems, University of Waterloo. The results of this study will contribute to Ms Courtney's honours thesis. If you have any questions specific to the research, please feel free to contact Dr. Shannon Majowicz at (519) 888-4567 ext. 31790 or smajowicz@uwaterloo.ca.

Purpose of the Study

To investigate the food safety knowledge, behaviours, and beliefs in undergraduate students at the University of Waterloo.

Procedures

If you agree to participate in this study, we would ask you to complete a 15-20 minute internet survey, which includes some background questions about you, questions about food safety practices and procedures, and questions surrounding your thoughts about food safety. Your participation in this study is completely voluntary. Your responses will remain anonymous, such that you are not asked for your name or any identifying information. It is important for you to know that any information that you provide will be confidential. All of the data will be summarized and no individual could be identified from these summarized results. Furthermore, the website is programmed to collect responses alone and will not collect any information that could potentially identify you (such as machine identifiers). You may stop the survey at any time or skip any questions that you prefer not to answer without penalty. This survey uses "Hosted in Canada Surveys" which is a Canadian company. If you prefer not to submit your data through Hosted in Canada Surveys, please contact one of the researchers so you can participate using an alternative method such as through an email or paper-based questionnaire. The alternate method may decrease anonymity but confidentiality will be maintained.

The information collected in this study will be kept for 30 years, as this is the anticipated length of the researcher's career, because we also want to look at how knowledge of food and health may change over future generations. However, no personally identifying information will be collected. The data will be accessible only by the project researchers and will be kept in a password protected database.

Ultimately, your participation will lead to a better understanding of food safety knowledge, behaviours, and beliefs among undergraduate students, and this information may be used to develop education programs focused on improving food safety among undergraduate students.

Remuneration for Participation

In appreciation of the time you have given to this study, you can enter your name into a draw for approximately 1 of 5 prizes. The prizes are a 50 dollar gift card to a location of your choice. Your odds of winning one of the prizes is based on the number of individuals who participate in the study. We expect that approximately 500 individuals will take part in the study. At

the end of the survey, you will be directed to a new site where you can choose to enter your email address for the gift card draw. Information collected to draw for the prize(s) will not be linked to the study data in any way, and this identifying information will be stored separately, then destroyed after the prize(s) have been provided. The amount received is taxable. It is your responsibility to report this amount for income tax purposes.

The draw will occur in April 2015.

Rights of Research Participants

You may withdraw your consent at any time and discontinue participation without penalty by not submitting your responses. You are not waiving any legal claims, rights or remedies because of your participation in this research study. This study has been reviewed and received ethics clearance through a University of Waterloo Research Ethics Committee. There are no known or anticipated risks associated with participation in the study. However, the final decision about participation in the study is ultimately yours. If you have questions regarding your rights as a research participant, contact Dr. Maureen Nummelin, Chief Ethics Officer at (519) 888-4567 ext. 36005 or maureen.nummelin@uwaterloo.ca.

Acceptance by Research Participant

Proceeding with the survey will indicate you accept our invitation to participate in the research project “Food Safety Knowledge, Behaviours and Beliefs in Undergraduate Students at the University of Waterloo” as described herein, and you are agreeing to participate in this study with the assurance that your responses will remain confidential.

There are 25 questions in this survey

Participant Consent

[]With full knowledge of all foregoing, I agree, of my own free will, to participate in this study. *

Please choose **only one** of the following:

- ○ I agree to participate.
- ○ I do not wish to participate (please close your web browser now).

Demographic Information

[]How old are you?

Only answer this question if the following conditions are met:
Answer was 'I agree to participate. ' at question '1 [A01]' (With full knowledge of all foregoing, I agree, of my own free will, to participate in this study.)

Please choose **only one** of the following:

- 18 years or younger
- 19 years
- 20 years
- 21 years
- 22 years
- 23 years or older

[]Please select your gender.

Only answer this question if the following conditions are met:
Answer was 'I agree to participate. ' at question '1 [A01]' (With full knowledge of all foregoing, I agree, of my own free will, to participate in this study.)

Please choose **only one** of the following:

- Male
- Female
- Other

[]Which faculty do you belong to?

Only answer this question if the following conditions are met:
Answer was 'I agree to participate. ' at question '1 [A01]' (With full knowledge of all foregoing, I agree, of my own free will, to participate in this study.)

Please choose **only one** of the following:

- Applied Health Sciences
- Arts
- Engineering
- Environment
- Mathematics
- Science

[]Which system of study do you belong to?

Only answer this question if the following conditions are met:
Answer was 'I agree to participate. ' at question '1 [A01]' (With full knowledge of all foregoing, I agree, of my own free will, to participate in this study.)

Please choose **only one** of the following:

- Co-op
- Regular

[]Do you currently work or volunteer in any of the following?

Only answer this question if the following conditions are met:
Answer was 'I agree to participate. ' at question '1 [A01]' (With full knowledge of all foregoing, I agree, of my own free will, to participate in this study.)

Please choose **all** that apply:

- [ ] A restaurant, deli, or other food service location
- [ ] A hospital
- [ ] A daycare or other place where you interact with children (e.g. Big Brothers/Big Sisters)
- [ ] A retirement home, nursing home, or long-term care facility
- [ ] Not applicable

[]If you checked any of the answers in (3), do you handle or prepare food in those places?

Only answer this question if the following conditions are met:
Answer was 'I agree to participate. ' at question '1 [A01]' (With full knowledge of all foregoing, I agree, of my own free will, to participate in this study.)

Please choose **only one** of the following:

- Yes
- No
- Not Applicable

[]Have you ever taken a course where you are taught how to prepare food or meals (e.g. high school classes, university classes, food handler certification)?

Only answer this question if the following conditions are met:
Answer was 'I agree to participate. ' at question '1 [A01]' (With full knowledge of all foregoing, I agree, of my own free will, to participate in this study.)

Please choose **only one** of the following:

- Yes
- No

[]How would you describe your ability to cook from basic ingredients?

Only answer this question if the following conditions are met:
Answer was 'I agree to participate. ' at question '1 [A01]' (With full knowledge of all foregoing, I agree, of my own free will, to participate in this study.)

Please choose **only one** of the following:

- I don't know how to cook
- I can only cook food when the instructions are on the box (like Kraft® Dinner)
- I can do the basics from scratch (like boil an egg or make a grilled cheese sandwich) but nothing more complicated
- I can prepare simple meals if I have a recipe to follow
- I can cook almost anything

[]Where do you currently live?

Only answer this question if the following conditions are met:
Answer was 'I agree to participate. ' at question '1 [A01]' (With full knowledge of all foregoing, I agree, of my own free will, to participate in this study.)

Please choose **only one** of the following:

- Traditional-style residence (V1, REV, St. Jerome's, St. Paul's, Renison, Conrad Grebel)
- Suite-style residence (MKV, UWP, CLV)
- Off campus
- At home

[]How often do you cook food or meals from basic ingredients?

Only answer this question if the following conditions are met:
Answer was 'I agree to participate. ' at question '1 [A01]' (With full knowledge of all foregoing, I agree, of my own free will, to participate in this study.)

Please choose **only one** of the following:

- At least once a day
- A few times a week
- A few times a month
- A few times a year
- Never

Food Safety Knowledge

[]Which procedure for cleaning kitchen counters is best?

Only answer this question if the following conditions are met:
Answer was 'I agree to participate. ' at question '1 [A01]' (With full knowledge of all foregoing, I agree, of my own free will, to participate in this study.)

Please choose **only one** of the following:

- Spray with a strong sanitizing solution
- Wash with a detergent, rinse, then wipe with a sanitizing solution
- Wipe with a sanitizing solution, then rinse with clean water and wipe dry
- Brush off any dirt or food pieces, then wipe with sanitizing solution

[]Which is the most hygienic way to wash your hands?

Only answer this question if the following conditions are met:
Answer was 'I agree to participate. ' at question '1 [A01]' (With full knowledge of all foregoing, I agree, of my own free will, to participate in this study.)

Please choose **only one** of the following:

- Apply sanitizer, run water, rub hands together for 20 seconds, rinse hands, dry hands, rub on an antiseptic hand lotion
- Apply soap, rub hands together for 20 seconds, rinse hands under water, dry hands, apply sanitizer
- Run water, moisten hands, apply soap, rub hands together for 20 seconds, rinse hands, dry hands
- Run water, moisten hands, apply sanitizer, rub hands together for 20 seconds, rinse hands, dry hands, rub on antiseptic hand lotion

[]Imagine that your electricity went off and the meat, chicken, and/or seafood in your freezer thawed and felt warm. What should you do?

Only answer this question if the following conditions are met:
Answer was 'I agree to participate. ' at question '1 [A01]' (With full knowledge of all foregoing, I agree, of my own free will, to participate in this study.)

Please choose **only one** of the following:

- Throw them away
- Cook them right away
- See how they smell or look before deciding what to do
- Immediately re-freeze until solidly frozen, then cook them

[]Which of the following is considered the most important way to prevent food poisoning?

Only answer this question if the following conditions are met:
Answer was 'I agree to participate. ' at question '1 [A01]' (With full knowledge of all foregoing, I agree, of my own free will, to participate in this study.)

Please choose **only one** of the following:

- Spray for pests in the kitchen area at least every week
- Rarely or never serve leftovers
- Keep foods refrigerated until it's time to cook or serve them
- Clean kitchen counters with sanitizing solutions weekly

[]If a family member is going to be several hours late for a hot meal, how should you store the meal to keep it safe until this person is ready to eat it?

Only answer this question if the following conditions are met:
Answer was 'I agree to participate. ' at question '1 [A01]' (With full knowledge of all foregoing, I agree, of my own free will, to participate in this study.)

Please choose **only one** of the following:

- Store it in the refrigerator and reheat it when the person is ready to eat it
- Store it in on the kitchen counter until the person is ready to eat it
- Store it in a cool oven until the person is ready to eat it
- Store it in a warm oven until the person is ready to eat it

[]All foods (except whole poultry) are considered safe when cooked to an internal temperature of:

Only answer this question if the following conditions are met:
Answer was 'I agree to participate. ' at question '1 [A01]' (With full knowledge of all foregoing, I agree, of my own free will, to participate in this study.)

Please choose **only one** of the following:

- 130 degrees Fahrenheit (54 degrees Celsius)
- 140 degrees Fahrenheit (60 degrees Celsius)
- 150 degrees Fahrenheit (66 degrees Celsius)
- 165 degrees Fahrenheit (74 degrees Celsius)

[]Which method is the best way of determining whether hamburgers are cooked enough?

Only answer this question if the following conditions are met:
Answer was 'I agree to participate. ' at question '1 [A01]' (With full knowledge of all foregoing, I agree, of my own free will, to participate in this study.)

Please choose **only one** of the following:

- Cut one to check the color of the meat inside
- Check the color of the juice to be sure it is not pink
- Measure the temperature with a food thermometer
- Check the texture or firmness of the meat
- Measure the length of time the hamburgers cook

[]To prevent food poisoning, how long should leftover foods be heated?

Only answer this question if the following conditions are met:
Answer was 'I agree to participate. ' at question '1 [A01]' (With full knowledge of all foregoing, I agree, of my own free will, to participate in this study.)

Please choose **only one** of the following:

- Until they are boiling hot
- Just until they are hot, but not too hot to eat right away
- Just until they are at least room termperature
- Reheating isn't necessary

[]Chilling or freezing eliminates harmful germs in food.

Only answer this question if the following conditions are met:
Answer was 'I agree to participate. ' at question '1 [A01]' (With full knowledge of all foregoing, I agree, of my own free will, to participate in this study.)

Please choose **only one** of the following:

- True
- False

[]Where do you think food safety problems are most likely to occur?

Only answer this question if the following conditions are met:
Answer was 'I agree to participate. ' at question '1 [A01]' (With full knowledge of all foregoing, I agree, of my own free will, to participate in this study.)

Please choose **all** that apply:

- Farms
- Food processing plants
- Warehouses
- Supermarkets
- Restaurants
- Homes
- Don't know

[]How long should leftovers be stored in the refrigerator?

Only answer this question if the following conditions are met:
Answer was 'I agree to participate. ' at question '1 [A01]' (With full knowledge of all foregoing, I agree, of my own free will, to participate in this study.)

Please choose **only one** of the following:

- 1 to 2 days
- 3 to 4 days
- Up to 7 days
- Base on look, smell, and taste of food

[]What are microorganisms?

Only answer this question if the following conditions are met:
Answer was 'I agree to participate. ' at question '1 [A01]' (With full knowledge of all foregoing, I agree, of my own free will, to participate in this study.)

Please choose **only one** of the following:

- Poisons that can contaminate our food and water
- Small living things that are too small to be seen with our eyes
- Small insects that we can see
- Large bugs that can land on our food and surfaces

Food Safety Beliefs and Attitudes

[]Please read each question, and circle the answer that is the closest match to your own opinion.

Only answer this question if the following conditions are met:
Answer was 'I agree to participate. ' at question '1 [A01]' (With full knowledge of all foregoing, I agree, of my own free will, to participate in this study.)

Please choose the appropriate response for each item:

	Strongly Disagree	Disagree	Neither Agree nor Disagree	Agree	Strongly Agree
I am interested in finding out how to avoid food poisoning	○	○	○	○	○
It is not worth my time to learn about preventing food poisoning	○	○	○	○	○
I like learning about how to keep my foods safe to eat	○	○	○	○	○
It is of little use to me to learn about how to prevent food poisoning	○	○	○	○	○
I would like to learn about how to prevent food poisoning	○	○	○	○	○
I am interested in finding out how to read nutrition labels	○	○	○	○	○
It is not worth my time to learn about how to read nutrition labels	○	○	○	○	○
I like learning about how to choose nutritious foods to eat	○	○	○	○	○
It is of little use to me to learn about nutrition labels	○	○	○	○	○
I would like to learn about how to read nutrition labels	○	○	○	○	○
I believe that I could get food poisoning	○	○	○	○	○
I have a chance of getting food poisoning	○	○	○	○	○
It is possible that I	○	○	○	○	○

could get food poisoning this year	○	○	○	○	○
I'm not someone who will get food poisoning	○	○	○	○	○
Food poisoning is not currently a big threat to my health	○	○	○	○	○
I do not worry about getting food poisoning from the food I eat	○	○	○	○	○
I am not concerned about getting food poisoning	○	○	○	○	○
Getting food poisoning is not a problem I worry about	○	○	○	○	○
I am concerned about getting food poisoning	○	○	○	○	○
I worry about getting food poisoning	○	○	○	○	○
Anyone can get sick with food poisoning, even me	○	○	○	○	○
Food allergies are not currently a big threat to my health	○	○	○	○	○
I am not concerned about food allergies	○	○	○	○	○
Food allergies are not a problem I worry about	○	○	○	○	○
I am concerned about food allergies	○	○	○	○	○
I worry about food allergies	○	○	○	○	○
There is little I can do to change my food preparation habits	○	○	○	○	○
I am worried that I may get sick if I eat a lunch that has sat out all day	○	○	○	○	○
I have no real control over the food I eat	○	○	○	○	○
I am confident that I can cook safe, healthy meals for myself and my family	○	○	○	○	○
I am worried about the environmental	○	○	○	○	○

impacts of the food I eat	○	○	○	○	○
Eating food that is produced locally is important to me	○	○	○	○	○
I’m not worried about how much food costs	○	○	○	○	○
Choosing the cheapest food option is most important to me	○	○	○	○	○
Choosing the most convenient food option is most important to me	○	○	○	○	○
Being able to cook safe, healthy meals is an important life skill	○	○	○	○	○
I am interested in finding out about how my food is grown	○	○	○	○	○
I am interested in knowing who grows my food	○	○	○	○	○
The Canada Food Guide is helpful to me	○	○	○	○	○
For me, the most important information on nutrition labels is the calories	○	○	○	○	○
For me, the most important information on nutrition labels is the sugar	○	○	○	○	○
I can eat whatever I want without it affecting me	○	○	○	○	○
I can drink high energy drinks like Red Bull without it affecting me	○	○	○	○	○
I am worried about caffeine poisoning from high energy drinks like Red Bull	○	○	○	○	○
I am not someone who will get caffeine poisoning	○	○	○	○	○

Food Preferences

[]These next questions are about food preferences and the ways you fix or prepare food. For each statement, select the answer that describes how you usually do things.

Only answer this question if the following conditions are met:
Answer was 'I agree to participate. ' at question '1 [A01]' (With full knowledge of all foregoing, I agree, of my own free will, to participate in this study.)

Please choose the appropriate response for each item:

	Strongly Disagree	Disagree	Neither Agree nor Disagree	Agree	Strongly Agree
I plan, or help plan, the meals in my household	○	○	○	○	○
Before preparing or handling food, I wash my hands with soap and warm running water	○	○	○	○	○
If I have a cut or sore on my hand, I cover it before preparing food	○	○	○	○	○
I wash the plate used to hold raw meat or chicken with hot soapy water before using it for anything else	○	○	○	○	○
I wash my hands with soap and warm running water after working with raw meat or chicken	○	○	○	○	○
I clean countertops with hot soapy water after preparing food	○	○	○	○	○
I refrigerate hot food within two hours of preparing and eating	○	○	○	○	○
I keep raw meat and chicken away from ready-to-eat foods like raw vegetables	○	○	○	○	○
I use a thermometer to check if meat or chicken has been cooked enough	○	○	○	○	○
I use a thermometer to check if leftovers have been reheated	○	○	○	○	○

enough					
I read nutrition labels to make decisions about the foods I choose	○	○	○	○	○
I read ingredient lists to make decisions about the foods I choose	○	○	○	○	○
I use an ice pack when I take my lunch to school	○	○	○	○	○
I use an ice pack when I take my lunch with me for day trips (like a trip to the beach)	○	○	○	○	○
After playing with a pet and before getting a snack, I wash my hands with soap and warm running water.	○	○	○	○	○
I use the Canada Food Guide to help me choose what to eat	○	○	○	○	○
When I cook or reheat meals, I use a microwave	○	○	○	○	○
When I cook or reheat meals, I use a regular oven	○	○	○	○	○
I eat food that has passed the “Best Before” date	○	○	○	○	○

If you would like more information about food safety, please go to:

http://www.regionofwaterloo.ca/en/safehealthycommunity/foodsafety.asp

http://www.phac-aspc.gc.ca/fs-sa/index-eng.php

To enter into the draw, please click on the following link:

Submit your survey.
Thank you for completing this survey.
