# Supplementary material for: Food safety knowledge of undergraduate students at a Canadian university: results of an online survey
Source: BMC Public Health. 2016 Nov 9;16:1147. doi: 10.1186/s12889-016-3818-y (PMC5103385; doi:10.1186/s12889-016-3818-y)
Supplement: Additional file 2: — Variables with Missing Data. (DOCX 51 kb) [file 12889_2016_3818_MOESM2_ESM.docx]

**Additional File 2. Variables with Missing Data**

| **Variable** | **Number missing out of 485 (% missing)** |
| --- | --- |
| Previous food course | 23 (4.74) |
| Gender | 8 (1.65) |
| System of study | 8 (1.65) |
| Age | 6 (1.24) |
| Frequency of cooking from basic ingredients | 6 (1.24) |
| Faculty | 5 (1.03) |
| Cooking ability | 5 (1.03) |
| Current living arrangement | 5 (1.03) |
| Current food handler | 4 (0.83) |
